# Custom prosthetic rehabilitation of ocular defects with a digital workflow

**DOI:** 10.1186/s41205-026-00338-6

**Published:** 2026-08-13

**Authors:** Siyi Wang, Qingwen Cai, Jin Pan, Xiaoyu Gu

**Affiliations:** 1 Department of Prosthodontics, Shanghai Ninth People’s Hospital, Shanghai Jiao Tong University School of Medicine, College of Stomatology, Shanghai Jiao Tong University, National Center for Stomatology, National Clinical Research Center for Oral Diseases, Shanghai Key Laboratory of Stomatology, Shanghai Research Institute of Stomatology, 500 Quxi Road, Shanghai, 200011 China; 2 Department of Dental Technology, Shanghai Ninth People’s Hospital, Shanghai Jiao Tong University School of Medicine, College of Stomatology, Shanghai Jiao Tong University, National Center for Stomatology, National Clinical Research Center for Oral Diseases, Shanghai Key Laboratory of Stomatology, Shanghai Research Institute of Stomatology, 500 Quxi Road, Shanghai, 200011 China

**Keywords:** Orbital prostheses, Ocular defect, Silicone, Negative mold, Digital technique

## Abstract

**Purpose:**

To use digital technologies, including high-resolution imaging, computer-aided design (CAD), and three-dimensional (3D) printing, to introduce a streamlined process by enabling rapid, reproducible, and cost-effective digital fabrication of orbital prostheses, thereby addressing the limitations of conventional manual prostheses, which remain labor-intensive, poorly reproducible, and often fail to achieve optimal marginal adaptation.

**Materials and methods:**

This study presented a digital workflow for silicone orbital prostheses fabrication, combining CT-derived 3D reconstruction, reverse engineering, and stereolithography to produce customized silicone prostheses with 3D-printed photocurable resin negative molds. Mimics 10.01 software was used for 3D reconstruction, and defect margin delineation and prosthesis design were performed using Geomagic Studio 2013 software.

**Results:**

The resulting prostheses demonstrated favorable facial symmetry, accurate skin color matching, excellent fit, and high wearing comfort, representing a promising approach to enhance the quality of life of our patients. This approach minimized reliance on extensive manual labor, facilitated archiving and reproduction, and offers satisfactory efficiency and esthetic outcomes, thereby providing a practical alternative for orbital rehabilitation.

**Conclusion:**

The digital workflow described herein established an efficient and systematic procedure for the fabrication of silicone orbital prostheses.

## Introduction

Loss of the entire ocular and orbital contents may result from various causes, including malignant tumors, severe trauma, congenital anomalies, or pathologic conditions. Beyond the obvious physical deformity, ocular loss often leads to functional impairments, such as difficulty in maintaining facial balance, as well as significant psychological and social consequences, including decreased self-esteem, social anxiety, and depression [1, 2]. Therefore, ocular–orbital prosthetic rehabilitation plays a vital role in restoring both appearance and confidence, ultimately improving the patient’s quality of life [3].

The fabrication of an orbital prosthesis is a complex and intricate process that presents a challenging journey. It requires careful consideration of patient-specific factors in determining the appropriate technique. The primary objective of such a prosthesis is to restore an anatomically acceptable structure and compensate for the cosmetic deficit resulting from ocular loss. Typically, orbital prostheses are fabricated following complete healing of the ocular socket, which generally occurs over a period of 2 to 4 months [4].

Among the available methods, adhesive-retained silicone orbital prostheses and custom polymethyl methacrylate (PMMA) ocular shells remain the most widely used approaches due to their simplicity, accessibility, and acceptable esthetic results. Traditional rehabilitation methods typically involve a conventional workflow of impression making, wax pattern sculpting, trial fitting, characterization, processing, and adhesive-based retention. These approaches are often time-consuming and labor-intensive, with limited reproducibility of natural contours and suboptimal marginal adaptation.

In patients with favorable bone quality—characterized by a stable bony coverage of the orbital defect, healthy margins, retentive undercuts, closed sinuses, and preserved eyebrow position—osseointegrated, implant-supported prostheses utilizing bar or magnetic attachments offer enhanced retention and positional stability [5]. While surgical reconstruction is considered the superior rehabilitation technique, it may not be feasible for patients with insufficient residual bone volume, complex anatomical alterations, or a history of radiotherapy after tumor resection, all of which complicate implant placement and osseointegration.

Maxillofacial prostheses can effectively camouflage mid-facial defects when surgical interventions are limited or contraindicated. Customized prostheses not only enhance facial aesthetics but also support the early psychosocial reintegration of affected individuals. Prosthetic rehabilitation offers several advantages, including procedural simplicity, minimal invasiveness, and the capacity for ongoing surveillance of disease recurrence, rendering it particularly suitable for frail or elderly patients [6, 7].

The integration of innovative technologies, including high-resolution imaging systems (e.g., computed tomography [CT]), computer-aided design (CAD) software for manipulation of complex three-dimensional (3D) digital datasets, and 3D printing, has the potential to reduce costs and address technical challenges that limit access to facial and orbital prostheses for patients worldwide [8]. The aim of this study was to present a simplified, cost-effective, and esthetically pleasing digital workflow for orbital prosthesis fabrication utilizing these digital technologies.

This clinical technique outlines the fabrication of customized orbital prostheses using computed tomography, reverse engineering, and three-dimensional (3D) printing technologies. CT-derived 3D reconstructions were employed to precisely capture the ocular defect. Defect margin delineation and prosthesis design were performed using Geomagic Studio 2013 software. Subsequently, a stereolithography apparatus (SLA) was used to produce a split negative mold, facilitating the fabrication of the silicone orbital prosthesis.

## Technique

**Fig. 1:**
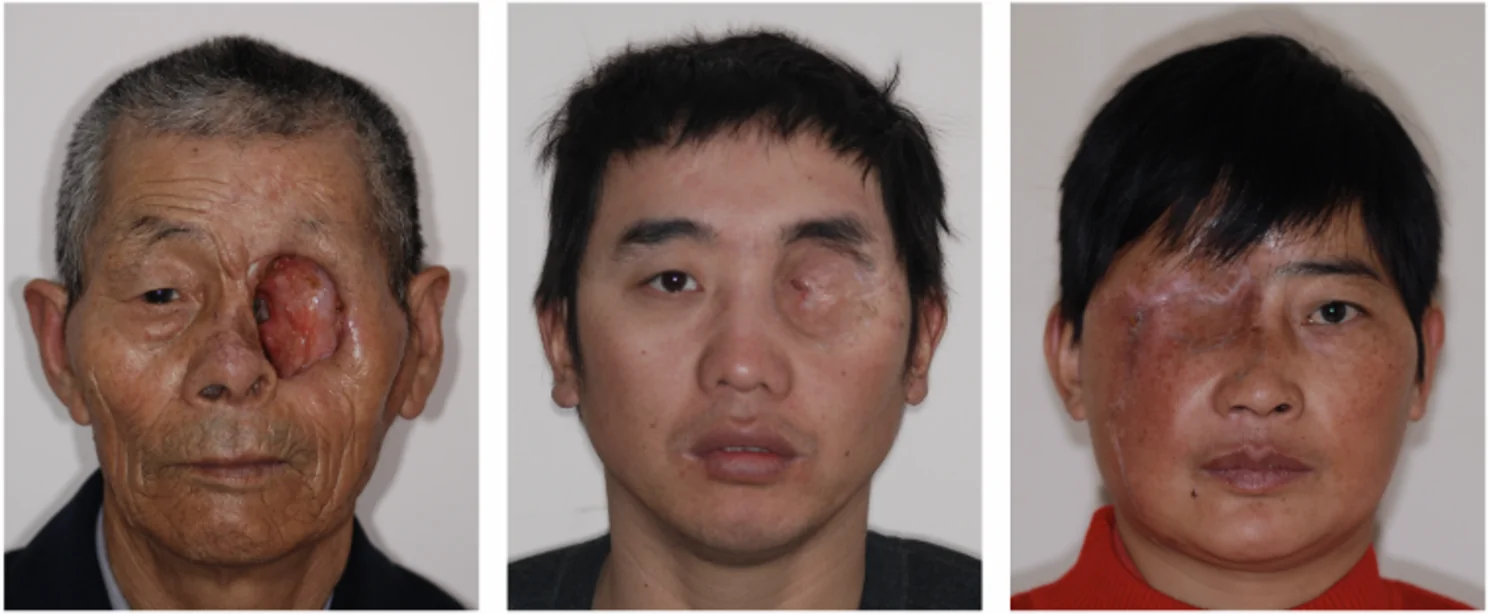
Three patients with ocular defects were enrolled in the study

Three patients with ocular defects presented at the Department of Prosthodontics, Shanghai Ninth People’s Hospital (Fig. 1). This study adheres to the tenets of the Declaration of Helsinki, and upon obtaining informed consent and the right to use the portrait, a digital workflow was employed for the partial restoration of ocular defects. One patient is selected to demonstrate this workflow with the following steps.

**Fig. 2:**
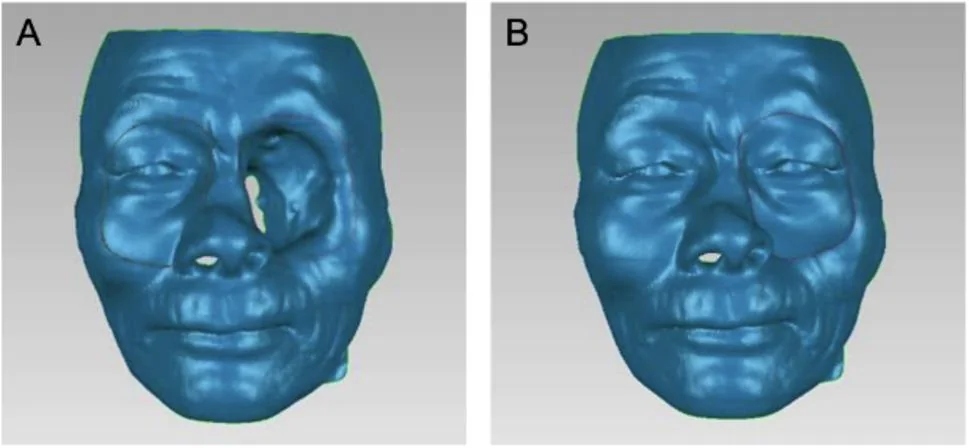
(**A**) First boundary mirror. (**B**) Second boundary mirror

**Fig. 3:**
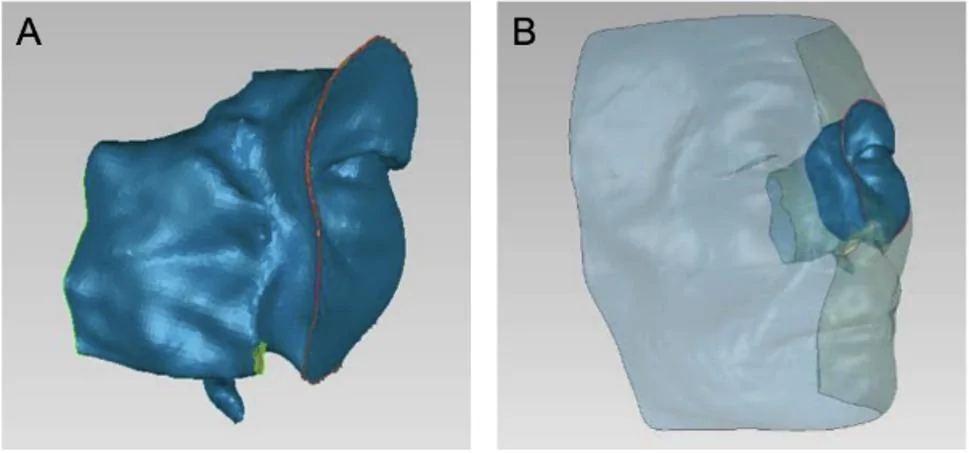
(**A**) Separate internal data of defect cavity. (**B**) Internal surface morphology and position of prosthesis

**Fig. 4:**
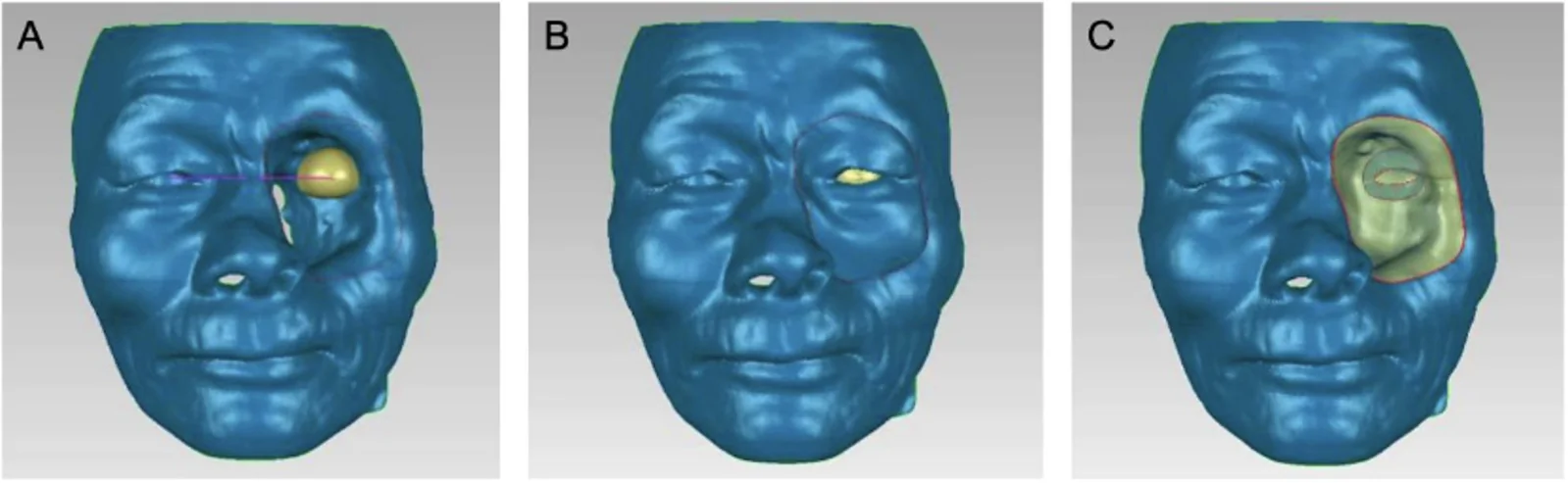
(**A**) Ocular shell localization process. (**B**) Formation of cleft eye morphology. (**C**) Forming the cavity of the ocular shell

**Fig. 5:**
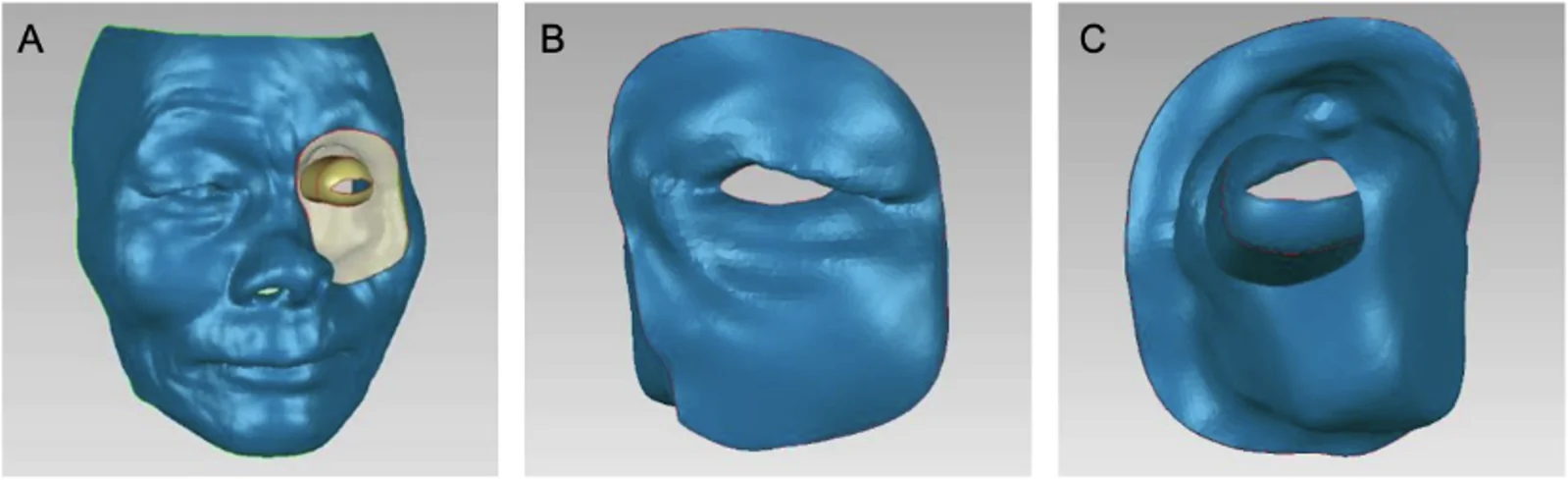
(**A**) Formation of ocular access channel. (**B**) Front view of the 3D morphology of prosthesis. (**C**) Tissue view of the 3D morphology of prosthesis

**Fig. 6:**
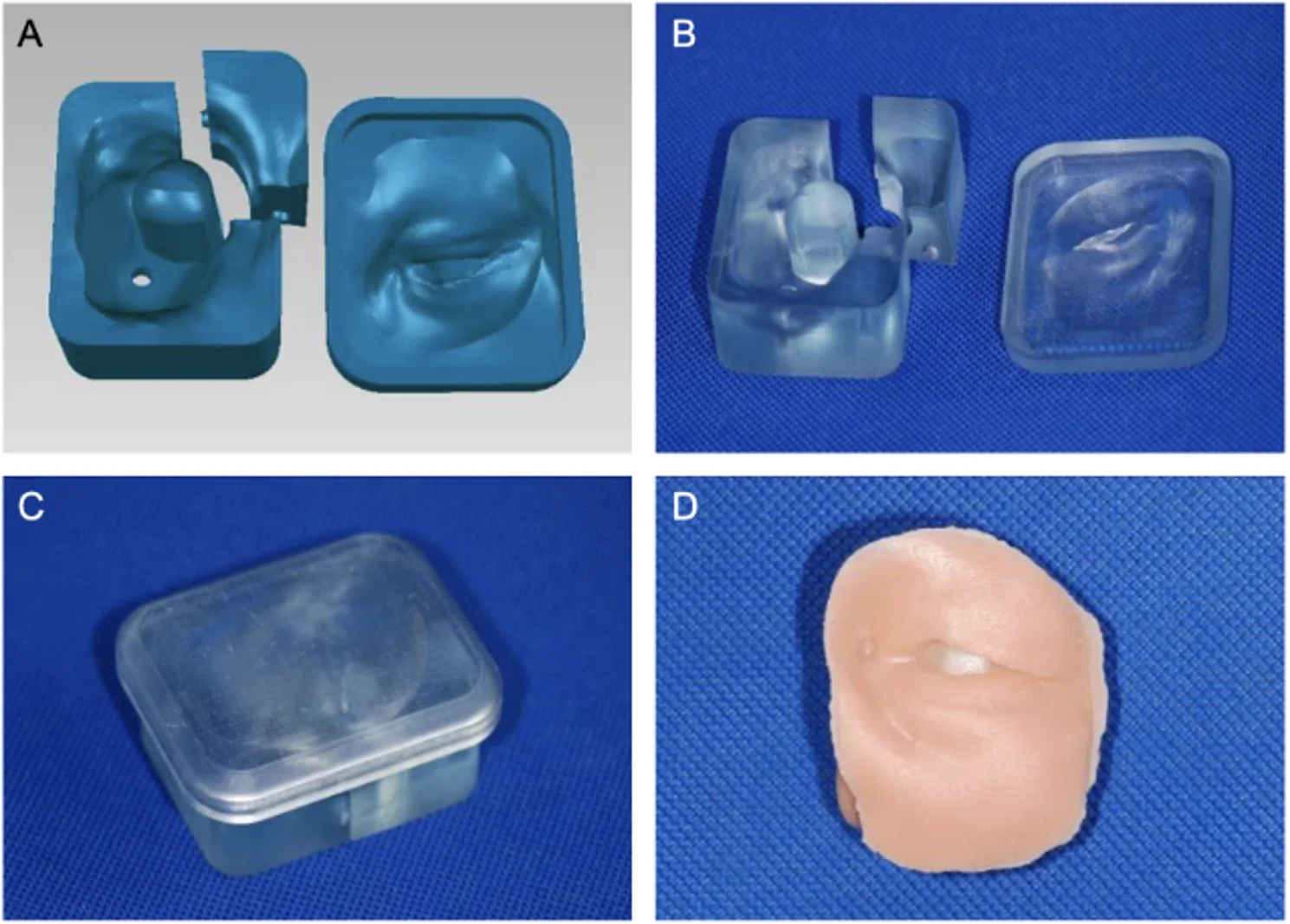
(**A**) Design a 3D negative mold for the prosthesis. (**B**) 3D print the negative mold with photocurable resin. (**C**) Assemble the negative mold. (**D**) The final silicone prosthesis

**Fig. 7:**
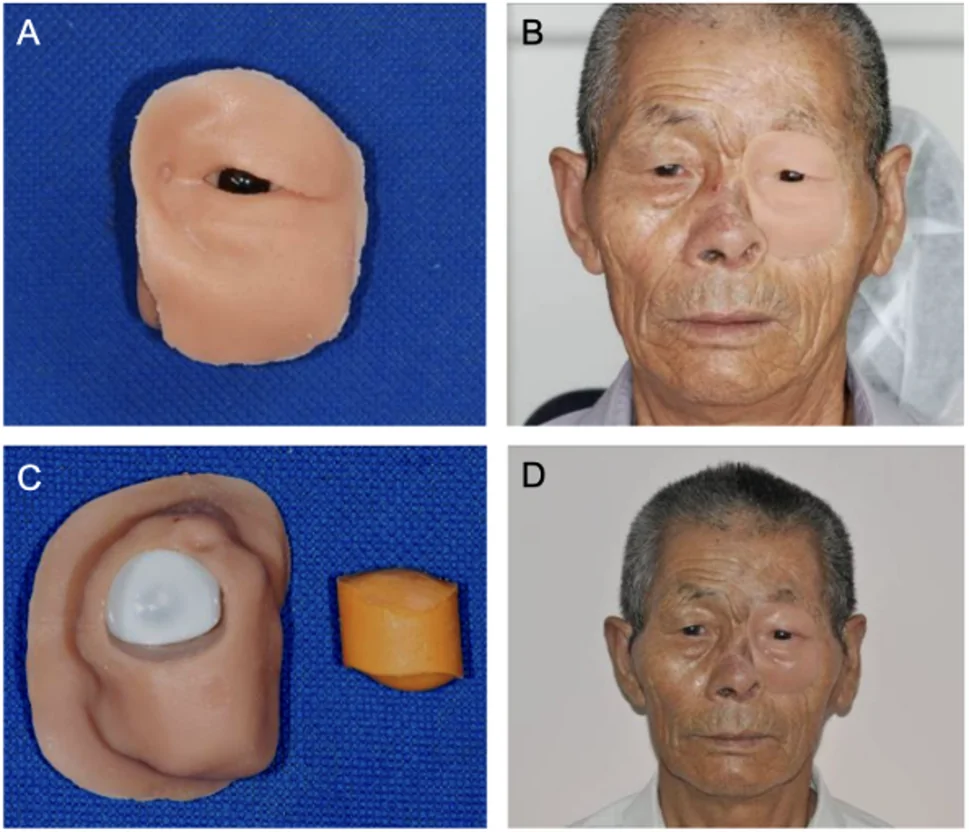
(**A**) Place the simulated ocular shell in position. (**B**) wear the prosthesis for the first time. (**C**) Seal the access channel of the ocular shell. (**D**) Final restorative effect

**Fig. 8:**
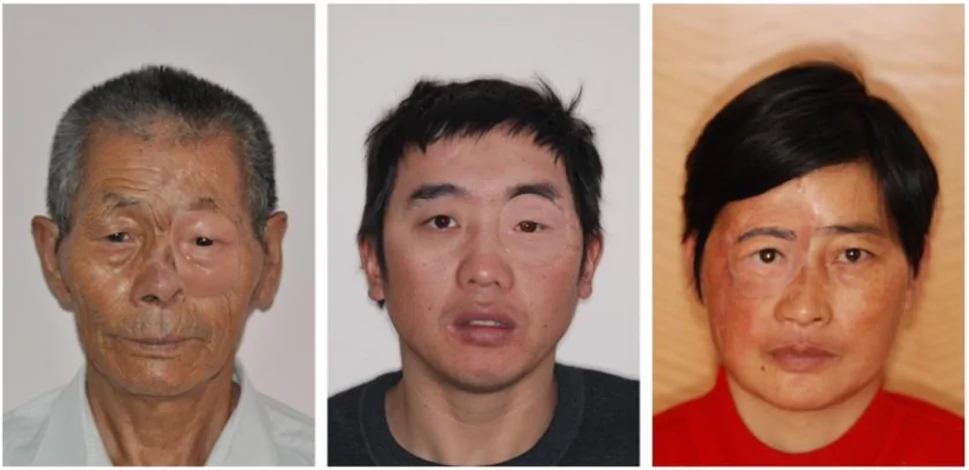
All three patients completed orbital prostheses rehabilitation

1. Perform spiral CT (Optima CT520Pro, GE, USA) scan of the orbital region (Pixel size 0.4–0.5 mm, layer spacing 1.00–1.25 mm). Obtain scanned data and store it in .DCM format. Use Mimics 10.01 software for 3D reconstruction (The predefined thresholds was set form − 700 Hu to 1958 Hu, which included the values of bone tissue and soft tissue) and save in .STL format.
2. Define the extension boundary. Import the CT reconstruction model into a reverse engineering software program (Geomagic Studio 2013 software) to form a 3D digital file of the defect. Define the extension boundary of the prosthesis on the 3D model to align with the natural direction of the patient’s skin texture and to transition smoothly onto the adjacent healthy soft tissue. Use the facial sagittal midplane as a reference, mirror the boundary line to the contralateral side and projected it onto the skin surface to determine the region for surface replication. Then mirror the triangular mesh data of that region back to the defect side, again use the sagittal midplane as the reference. After edge stitching along the defect boundary, refine the surface, relax tension points, and smooth the contours to form the external surface of the prosthesis. This step is referred to as the “secondary mirroring method” because the same reference plane was used for two consecutive mirroring operations during the design process (Fig. 2).

3. Design the external and internal surfaces. Design the external morphology of the prosthesis using the “secondary mirroring method”. Apply a 0.2-mm offset to the external surface to create a uniform edge thickness. Isolate the internal defect cavity data and design the internal surface of the prosthesis based on the established boundary. During this step, preserve the left nasal airway space and ensure that the posterior margin of the prosthesis do not extend into the pharyngeal region (Fig. 3).

4. Position the ocular shell. Select a prefabricated ocular shell by referencing the patient’s normal eye. Apply a wax marker to the center of the pupil of the finished ocular shell and scan the shell in 3D after applying a powder coating. Use the “object mover” function in the design software, position the ocular shell within the prosthesis so that the distance from the pupil center to the nasal midline match the previously measured distance on the contralateral, healthy side (Fig. 4A). The intersection of the ocular shell periphery and the eyelid contour on the prosthesis surface create a closed curve (Fig. 4B). Use Boolean operations to integrate the ocular shell data with the external surface, form a precise cavity for the ocular shell (Fig. 4C).

5. Create the ocular access channel. Design a boundary line along the transitional edge around the ocular shell and projected this boundary onto a plane to create a posterior ocular access channel (Fig. 5A). This channel allows the patient to remove and reinsert the ocular shell easily for cleaning and to make minor positional adjustments of the pupil to maintain symmetry with the contralateral eye. The final prosthesis design is shown in Fig. 5B and C.

6. Fabricate the prosthesis. Referring to the technical process of our previous article [9], design a 3D negative mold for the prosthesis. For this case, an auxiliary parting surface is designed, dividing the mold into three sections (Fig. 6A). 3D print the negative molds using Projet3510 P1us 3D printing equipment (3D Systems) with Stereo Lithography Apparatus (SLA) technology and photocurable resin material (resin material: VisiJet M3 Cristal, 3D Systems, USA; supporting material: VisJet S300, 3D Systems, USA) (Fig. 6B and C). Slowly inject silicone material (Multisil-Epithetik, bredent GmbH & Co KG, Deutschland) into the resin negative molds, press and fix the overall molds. Remove the silicone prosthesis from the mold for final detailing after a 16-hour cure at room temperature (Fig. 6D).

7. Adjust and deliver the prosthesis. Insert the ocular shell into the access channel of the prosthesis (Fig. 7A) and perform a clinical try-in to evaluate retention, stability, marginal adaptation, and the absence of interference with breathing or phonation (Fig. 7B). Adjust the ocular shell position as needed and fix it in place by injecting silicone impression material through the access channel (Fig. 7C). Affix commercially available synthetic eyelashes, typically obtained from beauty supply stores or pharmacies, to the prosthesis using clear silicone, identical to the material employed in prosthesis fabrication. After post staining of the prosthesis with hand using pigments (Extrinsic coloring kit, Factor II Inc, USA), deliver the prosthesis to the patient (Fig. 7D). Use water-based adhesive (g609 probond adhesive, Technovent, Britain) to fix the prosthesis. Instruct the patient to clean the internal surface of the prosthesis regularly and schedule follow-up evaluations.

8. Conduct the prostheses functioning evaluation. All three patients successfully completed orbital prostheses rehabilitation (Fig. 8). Based on the 20-item questionnaire for the Global Ocular Prosthesis Score (Table 1) [10], 20 questions were used to evaluate patients’ functional abilities and care, comfort, cosmesis, and psychosocial well-being. Each question was rated on a 5-point Likert scale, ranging from “fully agree” to “fully disagree.” Subdomain scores, calculated as the sum of the individual item scores within each domain, ranged from 5 to 25.

**Table 1:** 20-item questionnaire for the global ocular prosthesis score

	Fully disagree	Disagree	Neutral		Agree	Fully agree
**Functional abilities and care**						
1. I experience more balance problems since the loss of my eye.	5	4		3	2	1
2. I can easily pick up a glass from the table.	1	2		3	4	5
3. I was well informed by the ocularist about the treatment.	1	2		3	4	5
4. I bump into objects.	5	4		3	2	1
5. I worry about the health of my fellow eye.	5	4		3	2	1
**Comfort**						
6. I experience irritation when wearing my prosthesis	5	4		3	2	1
7. I can easily remove my prosthesis.	1	2		3	4	5
8. My prosthesis stays in place during sneezing and coughing.	1	2		3	4	5
9. I use wetting eye drops or ointment for my prosthesis.	5	4		3	2	1
10. I am concerned that the prosthesis can fall out.	5	4		3	2	1
**Cosmesis**						
11. My prosthesis looks larger than my other eye.	5	4		3	2	1
12. The iris (colored part) of my prosthesis looks the same as that of my other eye.	1	2		3	4	5
13. My prosthesis moves the same way as my other eye when looking to the right and left.	1	2		3	4	5
14. In the mirror, my face looks the same as before the loss of my eye.	1	2		3	4	5
15. The lower eyelid looks the same on both sides.	1	2		3	4	5
**Psychosocial**						
16. I feel uncomfortable looking people into the eyes.	5	4		3	2	1
17. People do not look at my face because of my eye.	5	4		3	2	1
18. I have stopped activities (social/sport/job/travel) because my eyes make me less able to concentrate.	5	4		3	2	1
19. I feel stressed because of my eyes.	5	4		3	2	1
20. I get fewer opportunities because of my eyes.	5	4		3	2	1

## Discussion

Orbital and oculofacial prostheses are regarded as the most practical and effective alternatives to local flap repositioning or microvascular free-flap reconstruction [11]. The fabrication of an orbital prosthesis, however, presents several challenges, including the need to develop a functional model without tissue compression, align the ocular component with the contralateral eye, replicate the form and structure of the periorbital tissues, establish the correct gaze and interlid aperture, and achieve an appropriate color match. Many of these challenges can be addressed through the application of digital technologies [12].

Digital design and three-dimensional (3D) printing represent a significant paradigm shift in maxillofacial prosthetics. High-resolution processing enables rapid and reproducible manufacturing while reducing manual labor and operator dependency. Moreover, a digital workflow facilitates archiving, straightforward replication, and long-term patient follow-up. Importantly, this approach minimizes the need for access to a highly skilled prosthetist, offering the potential for an efficient and standardized fabrication process for customized orbital prostheses, irrespective of a patient’s geographic location.

Silicones exhibit several advantageous properties, including biocompatibility, flexibility, moldability, chemical and physical inertness, and the capacity to incorporate both intrinsic and extrinsic pigments. In general, a silicone orbital prosthesis has an expected service life of approximately 1–2 years before replacement becomes necessary. Factors such as discoloration due to fluid absorption, skin oils, and debris deposition, material degradation, fraying of prosthetic margins, and changes in periorbital soft tissue contours can compromise aesthetics, fit, and overall usability [8]. In this study, orbital prostheses were fabricated by injecting silicone into 3D-printed negative molds, as few biocompatible silicone-based materials are currently available for direct 3D printing [13]. The negative molds produced through this workflow demonstrated sufficient rigidity, chemical compatibility, and stability to endure multiple iterations of silicone casting. Furthermore, because new molds can be generated directly from digitized data, the need for repeated patient scanning is eliminated.

Patient satisfaction following rehabilitation was evaluated. Overall, patients reported satisfactory outcomes in comfort and cosmesis. While their appearance and social participation were largely restored, some continued to experience diminished confidence and expressed concerns regarding potential disease recurrence. (Table 2). Both patients and their family members were instructed in the management of the silicone orbital prosthesis, including cleaning of the periabutment skin. In addition, written maintenance guidelines were provided (Table 3), with strong emphasis placed on adherence to these recommendations [11]. Follow-up visits were scheduled at 3-month intervals during the first year and at 6-month intervals thereafter.

**Table 2:** Patients’ scoring of 20-item questionnaire for the global ocular prosthesis

	Mean	Median
**Functional abilities and care**		
1. I experience more balance problems since the loss of my eye.	2.33	2
2. I can easily pick up a glass from the table.	4	4
3. I was well informed by the ocularist about the treatment.	5	5
4. I bump into objects.	4.33	4
5. I worry about the health of my fellow eye.	1.66	2
*Sub score*	*17.32*	*17*
**Comfort**		
6. I experience irritation when wearing my prosthesis	4	4
7. I can easily remove my prosthesis.	4	4
8. My prosthesis stays in place during sneezing and coughing.	3.66	4
9. I use wetting eye drops or ointment for my prosthesis.	2.33	2
10. I am concerned that the prosthesis can fall out.	3.66	4
*Sub score*	*17.65*	*18*
**Cosmesis**		
11. My prosthesis looks larger than my other eye.	4	4
12. The iris (colored part) of my prosthesis looks the same as that of my other eye.	3.66	4
13. My prosthesis moves the same way as my other eye when looking to the right and left.	1	1
14. In the mirror, my face looks the same as before the loss of my eye.	1	1
15. The lower eyelid looks the same on both sides.	3	3
*Sub score*	*12.66*	*13*
**Psychosocial**		
16. I feel uncomfortable looking people into the eyes.	3	3
17. People do not look at my face because of my eye.	3.66	4
18. I have stopped activities (social/sport/job/travel) because my eyes make me less able to concentrate.	4	4
19. I feel stressed because of my eyes.	2.33	2
20. I get fewer opportunities because of my eyes.	2.33	2
*Sub score*	*15.32*	*15*
*Total score*	*61.15*	*63*

**Table 3:** Maintenance care instructions for the patient

Maintenance care of a silicone prosthesis
1. Rinse the anterior and posterior surfaces of the prosthesis with lukewarm water. Since silicone color may lighten over time, handle and wipe the surfaces with care.
2. Thoroughly disinfect the inner surface that contacts the skin by carefully wiping with 70% ethanol.
3. After cleaning the prosthesis margins, apply adhesive partially using a cotton swab and attach the prosthesis to the skin.
4. Remove any residual adhesive from the skin and prosthesis edges using an appropriate adhesive remover.
5. Residual remover components can reduce adhesive strength during subsequent use. Therefore, after cleaning the prosthesis edges with adhesive remover, rinse again with water and disinfect with ethanol prior to storage.
6. Due to the inherent properties of silicone, the prosthesis can easily attract dust. Store the prosthesis in a protective case after washing and avoid prolonged exposure to direct sunlight.

Previous studies have shown that orbital prosthesis fabrication can be achieved through a variety of analogue, hybrid, and fully digital workflows, but most approaches still depend on either extensive manual sculpting or indirect data capture methods [14–16]. Compared with studies using facial scanning [8, 16], CT provides a more accurate representation of the defect anatomy and surrounding structures, thereby allowing maximal utilization of the defect undercut areas during prosthesis design. Moreover, most published techniques still rely on partial manual intervention, especially for ocular shell positioning, margin refinement, color characterization, and final clinical adjustment [3, 17, 18]. The present study contributes a more structured workflow by integrating CT-based reconstruction, defect-boundary-guided secondary mirroring, and digital ocular-shell positioning into a unified process designed for practical clinical use. Additionally, this workflow allows for fine postoperative adjustment and reproducible digital archive for future remanufacture.

Nevertheless, certain limitations persist. The cost of digital equipment and materials may limit widespread adoption, and additional training is required to integrate these techniques into daily practice. From a materials perspective, medical-grade silicone elastomers continue to be the standard for orbital prostheses. However, limitations in color stability and tear resistance remain a clinical challenge. Incorporating surface-treated fillers or high-performance pigments may further improve durability and long-term esthetics. Similarly, the development of new skin adhesives with improved peel strength and biocompatibility can enhance prosthesis longevity and patient confidence during functional activities.

In conclusion, our research combines the existing clinical principles with advanced digital technology to efficiently produce complex orbital prosthesis. The results show that the described production process can produce an orbital prosthesis with good facial symmetry, skin color matching, prosthesis suitability and wearing comfort, and ultimately become a promising way to improve the quality of life of patients. However, larger comparative studies with standardized outcome measures are needed to confirm the clinical advantages of this approach.

## Conclusion

Orbital prosthesis is a safe and widely accepted modality for the rehabilitation of orbital defects. The digital workflow described herein enabled the successful fabrication of customized silicone orbital prostheses with satisfactory fit and esthetic outcomes. The integration of CT-based reconstruction, secondary mirroring, digital ocular-shell positioning, and 3D-printed molds provided a practical and reproducible approach for orbital rehabilitation. By replacing most conventional steps with a rapid and precise automated process, this method alleviated the discomfort of patients and avoids soft tissue damage. Overall, this workflow demonstrated promising clinical feasibility for individualized orbital prosthetic restoration.

## Data Availability

The datasets used and/or analyzed during the current study are available from the corresponding author on reasonable request.
